# Inactivation of Selected Bacterial Pathogens in Dairy Cattle Manure by Mesophilic Anaerobic Digestion (Balloon Type Digester)

**DOI:** 10.3390/ijerph110707184

**Published:** 2014-07-14

**Authors:** Christy E. Manyi-Loh, Sampson N. Mamphweli, Edson L. Meyer, Anthony I. Okoh, Golden Makaka, Michael Simon

**Affiliations:** 1 Fort Hare Institute of Technology, University of Fort Hare, Alice Campus, Alice 5700, Eastern Cape Province, South Africa; E-Mails: smamphweli@ufh.ac.za (S.N.M.); emeyer@ufh.ac.za (E.L.M.); msimon@ufh.ac.za (M.S.); 2 Applied and Environmental Microbiology Research Group (AEMREG), Department of Biochemistry and Microbiology, University of Fort Hare, Alice Campus, Alice 5700, Eastern Cape Province, South Africa; E-Mail: aokoh@ufh.ac.za; 3 Department of Physics, University of Fort Hare, Alice Campus, Alice 5700, Eastern Cape Province, South Africa; E-Mail: gmakaka@ufh.ac.za

**Keywords:** anaerobic digestion, dairy manure, enteropathogens, viable counts, regression models, South Africa

## Abstract

Anaerobic digestion of animal manure in biogas digesters has shown promise as a technology in reducing the microbial load to safe and recommended levels. We sought to treat dairy manure obtained from the Fort Hare Dairy Farm by investigating the survival rates of bacterial pathogens, through a total viable plate count method, before, during and after mesophilic anaerobic digestion. Different microbiological media were inoculated with different serial dilutions of manure samples that were withdrawn from the biogas digester at 3, 7 and 14 day intervals to determine the viable cells. Data obtained indicated that the pathogens of public health importance were 90%–99% reduced in the order: *Campylobacter* sp. (18 days) < *Escherichia coli* sp. (62 days) < *Salmonella* sp. (133 days) from a viable count of 10.1 × 10^3^, 3.6 × 10^5^, 7.4 × 10^3^ to concentrations below the detection limit (DL = 10^2^ cfu/g manure), respectively. This disparity in survival rates may be influenced by the inherent characteristics of these bacteria, available nutrients as well as the stages of the anaerobic digestion process. In addition, the highest *p*-value *i.e.*, 0.957 for *E. coli* showed the statistical significance of its model and the strongest correlation between its reductions with days of digestion. In conclusion, the results demonstrated that the specific bacterial pathogens in manure can be considerably reduced through anaerobic digestion after 133 days.

## 1. Introduction

Public health and environmental hazards linked to livestock practices and their generated wastes are becoming a major challenge of local, national and global concern. This is because the animal wastes are most times not disposed of properly as they are employed as soil conditioners and fertilizers on agricultural land to improve soil quality for better crop yield [1]. In particular, Grewal *et al.* [2] highlighted that most dairy wastes are largely stored as a liquid in a lagoon, or as solid manure, packed into piles after which they are employed for land application. However, in the liquid form, manure has the potential to contaminate soil and surface water bodies through infiltration and/or runoff and present noxious odors. Livestock manure usually contains a diverse group of microorganisms, including bacteria, protozoa and viruses that can cause infections in humans and/or animals if not properly managed and contained [3]. Bacteria that have been well documented in manure include *Salmonella* sp., *E. coli* and *Campylobacter* species which are associated with human gastrointestinal infections, therefore manure is a potential source of contamination [4].

Clearly, to assess the threat posed by the aforementioned bacterial pathogens in the manure, the levels/survival rates of these pathogens need to be evaluated, which in turn determines the potential of the manure to cause contamination/infection [5]. This information can be obtained by viable plate count methods to monitor the duration of survival of pathogenic microorganisms shed in the animal wastes [6]. The survival rates of the bacterial pathogens reported in animal wastes can be influenced by the physico-chemical characteristics of the wastes and microbial competition. More elaborately, the level of these pathogens in manure is influenced by the physicochemical characteristic of the manure, which is dependent on the weather and soil conditions (*i.e.*, geographical location) [7] and also influenced by other factors such as age and species of the animal, diet and collection and storage facilities of the farms [8,9].

Nevertheless, the animal wastes can be treated via biological, physical and chemical methods in order to reduce or control the pathogenic microbes [10]. Moreover, the aforementioned pathogen control methods have been evaluated elsewhere and reports indicated that most mesophilic biological treatment processes are not likely to reduce pathogen levels by 90%–99%. However, to the best of our knowledge, no available data exist in literature on any of these methods being implemented by any dairy farm in the Eastern Cape Province of South Africa for the purpose of pathogen control. More especially, since Lutge and Standish [11] reported that very few on-farm digesters are available in South Africa and the biogas digesters were principally built and installed in the Western and Kwa-Zulu Natal provinces of the country [12]. In addition, the information about the potential environmental and health risks related to animal manure is obvious [13,14].

Against this background, this study sought to determine the reduction of *E*. *coli*, *Salmonella* and *Campylobacter* species by investigating their viable counts before, during and after mesophilic anaerobic digestion of dairy manure obtained from the Fort Hare Dairy Farm, Alice, Eastern Cape Province of South Africa for the purpose of reducing bacterial pathogens as one of the facets in the overall sanitization of the environment. In addition, regression models were developed to emphasize the relationship between the logarithmic bacterial count and the duration of digestion (retention time).

## 2. Materials and Methods

### 2.1. Experimental Set Up

**Figure 1:**
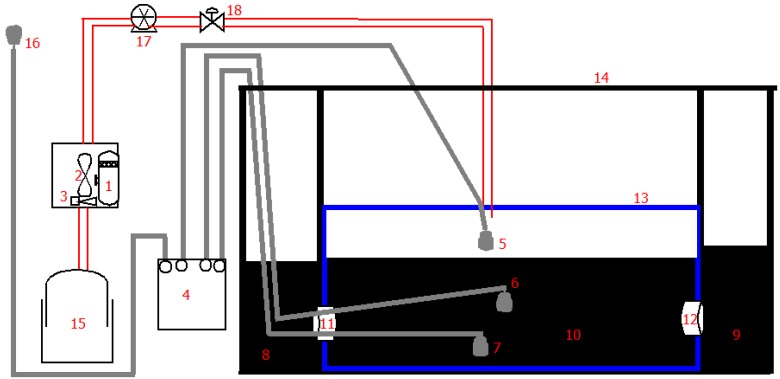
Designed layout of constructed balloon digester with the data acquisition system.

Notes: 1, Campbell data logger; 2, Gas analyser; 3, Flow transducer; 4, Hobo data logger; 5, Produce gas temperature sensor; 6, Intermediate slurry temperature sensor; 7, Bottom slurry temperature sensor; 8, Chamber inlet; 9, Chamber outlet; 10, Slurry trap inside balloon; 11, Slurry inlet; 12, Slurry outlet; 13, Balloon digester; 14, Insulation cover; 15, Gas collection chamber; 16, Ambient temperature sensor; 17, Pump; 18, Control valve

A balloon digester was enclosed within a concrete structure (8 m^3^) divided originally into three compartments: feeding tank of 83 cm height, 89 cm width and 95 cm length; bioreactor tank of 3.25 m height and 2 m width and lastly, the effluent tank (Figure 1). It was installed within the concrete structure because it is highly susceptible to physical damage and difficult to repair [12]. The balloon is the airtight chamber within which the anaerobic degradation of the dairy waste was conducted as both ends of the balloon were sealed with concrete to the influent chamber and the other to the effluent chamber. In addition, four temperature sensors (two sensors embedded at different levels in the slurry, one sensor at the biogas space and the last sensor within the concrete chamber housing the balloon digester (ambient) were externally connected to the Hobo U12, 4-channel data logger which was configured to log, the daily slurry, biogas and the ambient temperature profiles every 30 min. Also, a gas analyzer harboring methane and carbon dioxide gas sensors was connected through tubing incorporating a flow transducer. As the biogas flows through the tubing, the flow transducer measured the flow rate of the biogas into the gas analyzer, where the percentages composition of methane and carbon dioxide (important greenhouse gases) were detected.

Accordingly, the balloon digester was charged with a homogeneous mixture of dairy manure and water in the ratio 1:1 [15]. The mixing ratio was determined by the moisture content of the dairy manure procured from the Fort Hare Dairy Farm. The digester was batch operated under mesophilic conditions for six months.

### 2.2. Microbial Analysis

Total viable counts of bacterial colonies on solidified agar plates were used as a measure of the level of particular bacterial cells present in the manure. Initial bacterial counts in the original fresh manure were evaluated before the digestion process. In addition, during digestion, samples were withdrawn from the digester, every three, seven and fourteen day interval for continuous evaluation of bacterial counts. Each withdrawn sample was aseptically collected into sterile centrifuge tubes and transported on ice to the laboratory. Samples required for the determination of *Campylobacter* and *Salmonella* counts were introduced into the typtic soy broth before transportation to the laboratory [16].

Evaluation of the total bacterial counts was conducted as per the method of Poudel *et al.* [3]. Briefly, 1 g of each sample was serially diluted tenfold in 9 mL of sterile physiological saline. Dilutions from 10^−1^ to 10^−5^ were spread in triplicates on different microbiological media, including *Salmonella*/*Shigella* agar (Conda, Madrid, Spain), *E*. *coli* chromogenic agar (Conda), and blood free *Campylobacter* agar (mCCDA, Conda) to obtain total *Salmonella* sp., total *E*. *coli* and total *Campylobacter* sp. counts, respectively. All inoculated plates were incubated at 37 °C for 24 h, except that the plates for total *Campylobacter* counts were incubated at 42 °C under microaerophilic conditions provided by a gas generating kit (BR0038, Oxoid, Basingstoke, UK) for 24–48 h [17]. After incubation, the number of emergent colonies on each plate was counted taking into considerations their morphological and cultural characteristics [18,19]. Each value was recorded on a table and it represented the mean of triplicate spreading [20]. Of great significance, Sutton [21] recommended that where no colony was counted on the plate, the count should be recorded as less than the detection limit and the day of digestion was considered as the survival period of the pathogen. As indicated in this study, 10^−1^ (1:10) dilution produced no colonies on plates, the number of counts was <10 cfu/g. Furthermore, after the 10-fold dilution, we plated just 100 μL (not a full mL) of 1:10 dilution. In effect, an additional 10^−1^ dilution (100 µL/1000 µL).Therefore, the total effective dilution was 10^−1^ × 10^−1^ = 10^−2^. Hence, the detection limit was 10^2^ cfu/g manure.

### 2.3. Statistical Analysis

Data were analyzed and a linear regression model was developed between log bacterial load and days of anaerobic digestion using Matlab statistical software package (Student version, R2012a, The MathWorks, Inc., Natick, MA, USA).

## 3. Results

**Figure 2:**
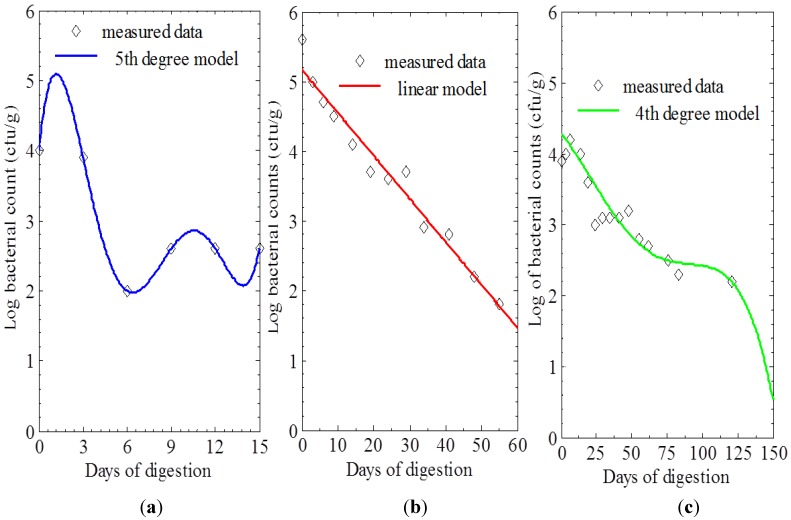
(**a**) Reduction of *Campylobacter* sp. counts with retention time. (**b**) Reduction of *E. coli* counts with retention time. (**c**) Reduction of *Salmonella* sp. counts with retention time.

Clearly, *Campylobacter* sp., *Escherichia coli* and *Salmonella* sp. were present in the manure with initial viable counts of 10.1 × 10^3^, 3.6 × 10^5^ and 7.4 × 10^3^ cfu/g, respectively, whose equivalence in logarithmic values were 4, 5.6 and 3.9 cfu/g, respectively. These bacteria were identified and confirmed by their morphological and culture characteristics. In addition, the viable counts of the bacteria in the manure before, during and after anaerobic digestion were converted to logarithmic values. These logarithmic values were plotted against retention time in days as shown in Figure 2a–c to reveal their relationship. The bacterial load (log viable counts) of *Campylobacter* sp., *E*. *coli* and *Salmonella* sp. decreased with an increase in the number of days from 4, 5.6, and 3.9 to <detection limit, respectively, through a period of 18, 62 and 133 days, respectively. These different numbers of days represented the days in which no growth was observed from the 10^−1^ dilution of the sample used for the isolation of each of the bacterium. The survival rate in days was therefore in the sequence; *Campylobacter* sp. < *E. coli* < *Salmonella* sp.

On the other hand, a 1 log reduction (90%) of *E*. *coli* and *Campylobacter* sp. was obtained during the period of digestion whilst *Salmonella* sp. were reduced by 2 log (99%) between day 9 and 14 and 1 log reduced throughout the rest of the process.

The relationship between log bacterial counts and the days of digestion are shown in Table 1. The regression models were developed wherein the last entry on the data set (<100 cfu/g, the detection limit) was excluded in order to maintain data integrity. These models indicated the kind of relationship and the highest *p*-value of 0.957 noted the statistical significance/degree of validity presented by the regression model incorporating the log reduction of *E. coli* with days of digestion.

**Table 1:** Regression model of log bacterial count with retention time (days).

Bacteria	Log of Count	X^5^	X^4^	X^3^	X^2^	X	Constant	*R*^2^	*p*-value
ERN	Y	0	0	0	0	−0.61	5.2	0.976	0.957
SMN	Y	0	−4.5 × 10^−8^	1 × 10^−5^	−5.1 × 10^−4^	−0.023	4.3	0.960	0.773
CPL	Y	3.8 × 10^−3^	−0.015	0.21	−1.2	2.1	4	0.991	0.723

Notes: ERN, *Escherichia coli*; SMN, *Salmonella* sp.; CPL, *Campylobacter* sp.; Y = log the bacterial count and X = days of digestion.

## 4. Discussion

Theoretically, under normal circumstances and favorable conditions, a bacterium tends to thrive in the environment where it occurs by first adapting to the environment and subsequently growing, multiplying or dividing by binary fission. Later, when the growth of these bacterial cells becomes limited, they stop dividing until they eventually die, causing a decline in their population due to lack of space, exhaustion of nutrients and accumulation of toxic waste products. Factors such as moisture, temperature, and pH, nutrients (both macro- and micronutrients) are necessary for microbial survival [22]. However, this pattern of growth is often not represented practically in the environment. Specifically, the decay rate of viable bacterial in a biogas plant can be influenced by many factors including temperature, treatment time, pH, volatile fatty acids, batch or continuous feeding process, bacterial species and available nutrients [23].

For farm-based purposes, we investigated the significance of using an anaerobic digester to sanitize dairy cattle manure prior to further usage. This was achieved by monitoring the fate of selected zoonotic pathogens such as *Campylobacter* sp., *Salmonella* sp. and *Escherichia coli* by a viable plate count assay. The viable counts revealed the viability of these bacteria with time and ranged from 10.1 × 10^3^, 3.6 × 10^5^, and 7.4 × 10^3^ to concentrations below the detection limit (<100 cfu/g), respectively. We noted that the survival rates of the aforementioned pathogens were in the order; *Campylobacter* sp. (18 days) < *Escherichia coli* (62 days) < *Salmonella* sp. (133 days) as shown in Figure 2a–c. However, this contradicts the finding of Strauch [7] who reported that the inactivation of pathogens in manure is reliant on the initial level of these pathogens present. Accordingly, *E*. *coli* with the highest concentration of 3.6 × 10^5^ cfu/g, determined in manure before the anaerobic digestion treatment, was expected to take the longest time to be inactivated. Contrarily, in this study, *Salmonella* sp. with the least concentration of 7.4 × 10^3^ cfu/g had the longest survival period.

Regarding the log reduction of these enteropathogens during their survival periods, a 1log reduction of both *E*. *coli* and *Campylobacter* species was achieved, indicating a 90% killing rate of these bacterial cells during digestion. Contrarily, the decay rate of *Salmonella* cells was not consistent; a 2 log reduction of these cells occurred between day 9 and 14 and a 1 log reduction throughout the rest of the process representing a killing rate of 90% to 99% during the anaerobic degradation process. Despite the magnitude of the log reduction, these small reductions in the prevalence or levels of these zoonotic pathogens in manure being treated anaerobically would significantly lower the risk of pathogen dissemination through land application/spreading of manures as large quantities of wastes are often generated [5]. In addition, this method of treatment generated biogas; an alternative energy source which could be used for heating and or converted to electricity by combined heat and power plants for other purposes. Moreover, the greenhouse gases (methane and carbon dioxide) were confined within the digester thus prevents global warming [24].

Although, we measured some physicochemical characteristics of our manure sample that are relevant for anaerobic digestion, and which also support the growth of these bacteria, literature has it that cattle manure is well balanced nutritionally with a C:N ratio of between 20 and 30 and is a good source of nitrogen; all of which are necessary for the growth of anaerobic microorganisms [25]. In addition, Sahlström [23] in his findings noted the variation in the survival rates between bacteria and attributed it to the differences in bacterial species (type) and available nutrients. Consequently, as shown in Figure 2a–c, the duration of survival of these bacteria *(i.e.*, 18, 62 and 133 days for *Campylobacter* sp., *E*. *coli* and *Salmonella* sp., respectively) could be influenced by the characteristics endowed to each bacteria even though they belong to the same domain, bacteria. It is obvious that being classified under different genus and species inferred they might possess different phenotypes and genotype characteristics, thus they might vary in the environmental requirements necessary for their growth [26,27]. Furthermore, the growth of these bacteria could also be affected by the different stages that are involved in the degradation process of the organic matter under anaerobic conditions. This could simply be explained by the fact that the decomposition of any organic material entails different degradation steps that requires microorganisms which may be specific for each stage, thus resulting in varying end products throughout the process [27].

More elaborately, the trend in the survival rate might possibly be explained by the fact that *Campylobacter* sp. is microaerophilic in nature [28] thus subjecting these cells under strict anaerobic conditions would cause them to die out quickly after a while (in this case 18 days) as compared to the other bacterial cells that are facultative in nature (*i.e.*, can survive both in the presence and absence of oxygen). Moreover, the early phases of anaerobic degradation process produce CO_2_ and a decrease in pH (due to the level of volatile fatty acids) [29]. This may suggest that *Campylobacter* cells were most probably sustained under such conditions and eventually die out as the process progresses. Not withstanding, Bui *et al.* [30] reported that the ability of *Campylobacter coli* to survive in manure and the capability to cause human infection are not clearly comprehended.

Furthermore, *Escherichia coli* and *Salmonella* sp. belong to the group *Enterobacteriaceae* that are responsible for the deconstruction of biomass during the first step in the bioconversion of carbohydrates to methane, as active fermenters that are anaerobic in nature [31,32,33]. This may suggest that the prevailing anaerobic conditions under which decomposition was conducted, supported these organisms for a further length of time as opposed to *Campylobacter* species. However, the overall results of the rate of killing of the bacteria (log reduction) contradict earlier reports by Côté *et al.* [20], Poudel *et al.* [3] and Harrison *et al.* [34] who reported the reduction of these enteropathogens by 97.94% to 100%, 99.999% and 99% to 100%, respectively.

These contradictory results are expected because the metabolic activities of specific organisms during anaerobic digestion could be influenced by the chemical composition of the wastes, environmental factors (temperature), digester operating conditions (batch or continuous mode) as well as the type of digester used [23,35]. Moreover, the physicochemical properties of wastes are dependent on the weather and soil conditions that influence the feed (vegetation) of these animals and this might vary from one geographical location to the other added to the fact that the overall microbial species also vary with farm practices and the aforementioned environmental conditions.

In addition, from the above Figure 2a–c, the regression lines were the generated models. From these, it can be depicted that the log of bacterial counts reduced with an increase in digestion time (number of days). However, the rate of reduction of the bacteria was in the order *Campylobacter* sp. < *E. coli* < *Salmonella*; even though, it was reported that the inactivation of microbial pathogens is subject to the initial counts of the specific microbes present in the wastes [7]. Furthermore, the equations for the different models are as shown in Table 1 and these regression models were used to forecast the log bacterial count reduction with time. The linear regression model showing the log reduction of *E*. *coli* with days of digestion has the highest *p*-value of 0.957 thus it is statistically significant indicating that the experimental data fits well into the model and 97.6% of the data variability can be interpreted by its regression model therefore, a higher validity of the model.

## 5. Conclusions

Anaerobic digestion technology was shown as a promising technology for the reduction of bacterial pathogens occuring in dairy manure as one of the facets in the overall process of sanitizing the environment for better public health safety. The microbial load was grossly reduced and the reduction of bacterial pathogen depended on the days of digestion. This indicates the need for biogas digesters on farms to recover biogas, a cheaper source of energy from the readily available animal manure and in addition aid in sanitization. Furthermore, the information generated by the interpretation of data recorded in this study, provides the basis for the development of guidelines essential for good and suitable plans for manure handling by farmers as they can be correlated with the farm practices thereby helping the farmers in proper management.
